# Dual Effects of Lipid Metabolism on Osteoblast Function

**DOI:** 10.3389/fendo.2020.578194

**Published:** 2020-09-23

**Authors:** Nathalie S. Alekos, Megan C. Moorer, Ryan C. Riddle

**Affiliations:** ^1^Department of Orthopaedic Surgery, Johns Hopkins University School of Medicine, Baltimore, MD, United States; ^2^Baltimore Veterans Administration Medical Center, Baltimore, MD, United States

**Keywords:** osteoblast, fatty acid metabolism, dyslipidemia, bone mass, lipoproteins

## Abstract

The skeleton is a dynamic and metabolically active organ with the capacity to influence whole body metabolism. This newly recognized function has propagated interest in the connection between bone health and metabolic dysfunction. Osteoblasts, the specialized mesenchymal cells responsible for the production of bone matrix and mineralization, rely on multiple fuel sources. The utilization of glucose by osteoblasts has long been a focus of research, however, lipids and their derivatives, are increasingly recognized as a vital energy source. Osteoblasts possess the necessary receptors and catabolic enzymes for internalization and utilization of circulating lipids. Disruption of these processes can impair osteoblast function, resulting in skeletal deficits while simultaneously altering whole body lipid homeostasis. This article provides an overview of the metabolism of postprandial and stored lipids and the osteoblast's ability to acquire and utilize these molecules. We focus on the requirement for fatty acid oxidation and the pathways regulating this function as well as the negative impact of dyslipidemia on the osteoblast and skeletal health. These findings provide key insights into the nuances of lipid metabolism in influencing skeletal homeostasis which are critical to appreciate the extent of the osteoblast's role in metabolic homeostasis.

## Introduction

Development of the mammalian skeleton and maintenance of its structure for the life of the organism requires the coordinated actions of two specialized cells. Osteoclasts, large multinucleated cells that are derived from the monocyte/macrophage lineage of hematopoietic cells, are responsible for bone resorption. After attaching to an exposed bone surface, osteoclasts acidify a resorption lacuna to dissolve the mineral fraction of bone and then secrete proteolytic enzymes that degrade the organic matrix component (1). During the resorption process, growth factors trapped within bone matrix are released and trigger the recruitment of osteoblasts responsible for new bone formation (2, 3). Derived from mesenchymal stem cells present in the bone marrow stroma, these cells are characterized by their cuboidal shape and abundance of rough endoplasmic reticulum necessary for the production of the collagen-rich bone matrix (4). After building a packet of bone most osteoblasts will die by apoptosis, but small fractions will either become encapsulated within the bone matrix and fulfill regulatory functions as osteocytes or dedifferentiate and line bone surfaces. Known as bone remodeling, this process prevents the accumulation of old or damaged bone that may lead to fracture. In humans, peak bone mass is reached during the second decade of life as a result of net bone accrual during childhood, when bone formation exceeds resorption and osteoblasts and osteoclasts act on different bone surfaces to maintain the overall shape of bones during longitudinal growth (known as modeling). A balance between formation and resorption then occurs in early adulthood. However, with advancing age or as a result of numerous endocrine pathologies, an acceleration of osteoclastic activity leads to bone loss as osteoblastic activity is unable to keep pace. As bone mass decreases and structure integrity deteriorates, the risk of fracture increases (5, 6).

The tremendous economic impact of osteoporotic fractures (7–9) and development of comorbidities after fracture (10–12) highlight the need to understand the genetic, cellular, and endocrine mechanisms that influence bone mass. With the renewed interest in intermediary metabolism in cancer (13–15) and the recognition that bone is not merely a structural organ acting as a reserve of minerals but also an endocrine organ that can influence systemic metabolism (16–21), research in the field of skeletal biology has coalesced over the last few years on the contributions of cellular metabolism to osteoblast function and bone formation. The field reasoned that if bone contributes to the regulation of metabolic homeostasis through the release of osteocalcin and other hormones (16, 21), then the availability of nutrients must be critical to osteoblast function. Indeed, hierarchical analysis of energy requirments of cellular function (22) suggest that the bone remodeling process is energy intensive due to the synthesis of large extracellular matrix proteins and the necessity of concentrating mineral ions for hydroxyapatite crystal formation. Evidence from both the laboratory and the clinic supports this hypothesis as caloric restriction during gestation or during postnatal life strongly influences the trajectory of both the accrual and the maintenance of bone mass (23–25). Additionally, an increase in oxidative phosphorylation and the abundance of mitochondria appears to be a requirement for the differentiation of osteoblasts from marrow stem cells (26–29).

Osteoblasts harvest energy from a number of fuel molecules. Studies performed more than 50 years ago first highlighted the avidity of osteoblasts for glucose. Isolated osteoblasts or bone tissue explants from mice, rats, rabbits, and humans all used glucose to produce lactate even under aerobic conditions (30–34). More contemporary studies indicated that glucose acquisition is mediated by glucose transporter-1 (35) and that metabolic programming of glucose utilization is adjusted according to the stage of differentiation (28, 36). Cells of the osteoblast lineage also consume a significant amount of glutamine which is required for skeletal stem cell specification, can be catabolized by the tricarboxylic acid cycle to generate ATP, and serves as a regulatory signal to maintain endoplasmic reticulum health during stages of heightened protein synthesis (37, 38).

While lipid metabolism yields significantly more ATP than glucose or glutamine catabolism, its role in osteoblast function remains more controversial. Recent studies have highlighted the importance of fatty acid catabolism for normal bone formation (39, 40), but detrimental effects of lipids on osteoblast performance are also well-known (41, 42). In this review, we discuss the dual effects of lipids on osteoblast function and the maintenance of bone mass and strength. We provide a brief overview of the trafficking and metabolism of lipids in target tissues like bone. We then describe studies which highlight the importance of fatty acids metabolism for the accrual of bone mass and the mechanisms that regulate fatty acid utilization. Finally, we discuss the effects of dyslipidemia on osteoblast function and the potential for this condition to desensitize osteoblasts to anabolic signals.

## Overview of Lipid Metabolism

**Figure 1:**
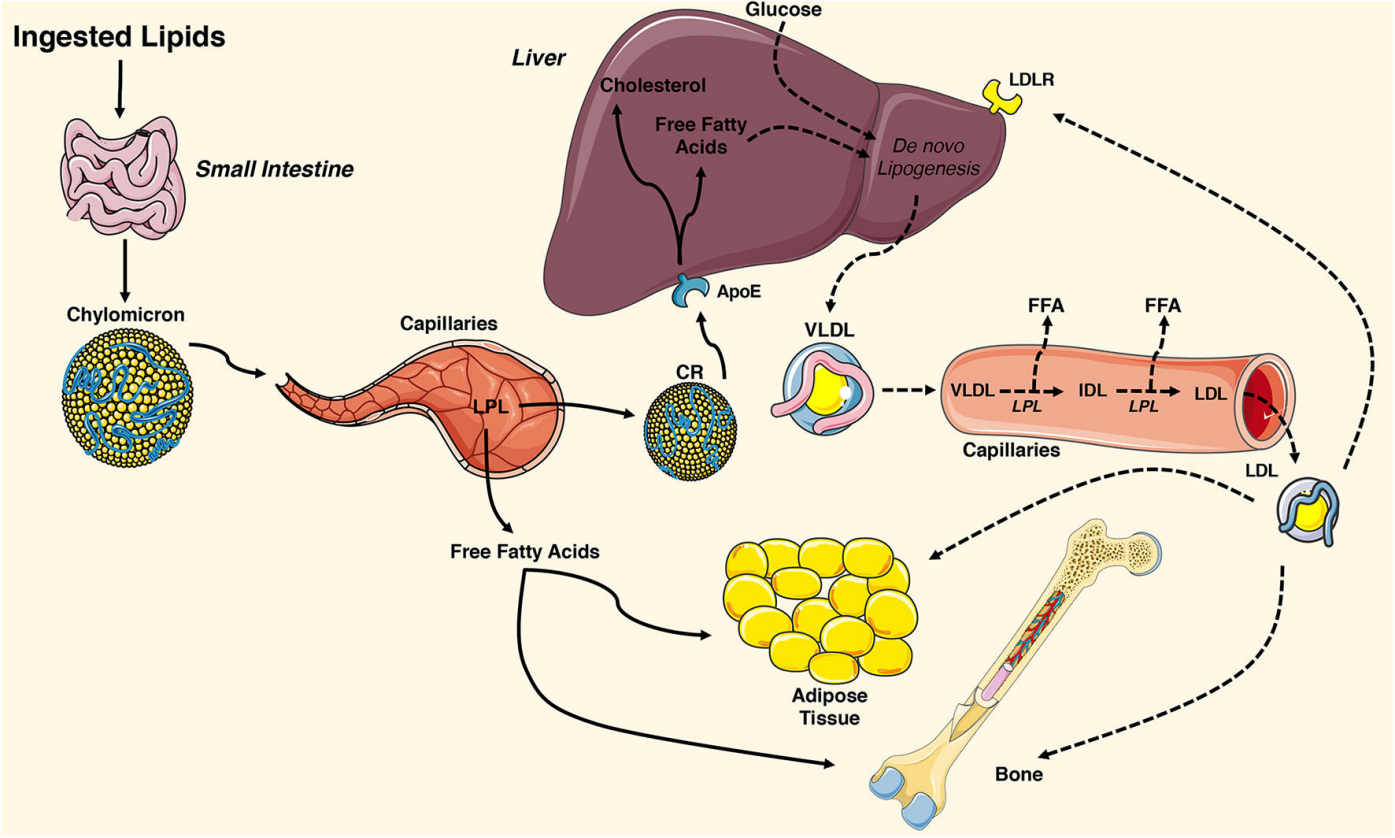
Overview of tissue-targeted lipid metabolism. Ingested lipids are broken down in the intestinal lumen and internalized by enterocytes of the small intestine. The water insoluble triglycerides and cholesterol are repackaged into chylomicrons and travel through the lymphatic system and into the circulatory system where they engage lipoprotein lipase (LPL) on the surface of capillary endothelial cells. The hydrolyzed triglycerides result in release of free fatty acids that are taken up by adipose tissue and the skeleton. The remaining chylomicron remnants (CR) are cleared by the liver via the apolipoprotein E (ApoE) receptor. CR-derived cholesterol and free fatty acids and circulating glucose are used for *de novo* lipogenesis, generating ATP for the liver, or repackaged into very low-density lipoproteins (VLDL). VLDL particles are released into the circulation where they engage LPL and release free fatty acids, which are also available for uptake. The remaining low-density lipoprotein (LDL) are internalized by cells expressing the low-density lipoprotein receptor (LDLR) including adipocytes and osteoblasts. This figure was created using Servier Medical Art image templates under a Creative Commons Attribution 3.0 Unported License.

The lipid molecules that support cellular metabolism are primarily derived from three sources: ingested fat, lipoproteins produced by the liver, and non-esterified fatty acids released by white adipose tissue (Figure 1). Postprandial triglycerides and cholesterol esters are broken down in the intestinal lumen by cholesterol esterases, pancreatic lipases, and bile salts. These molecules are then taken up by the enterocyte of the small intestine, re-esterified, and packaged with lipid-soluble vitamins, and apolipoproteins into chylomicrons. Chylomicrons enable water-insoluble fats and cholesterol to move through the lymphatic system and into the circulatory system. Engagement of the chylomicron by lipoprotein lipase (LPL) on capillary endothelium results in the hydrolysis of triglycerides and the delivery of fatty acids to target tissues (43, 44). The chylomicron remnants containing cholesterol and apolipoproteins are then cleared by the liver (45).

In healthy individuals, the liver exhibits a nearly constant lipid flux. Chylomicron remnants and free fatty acids are taken up by the liver, while a portion of the circulating glucose taken up by the organ is used for *de novo* lipogenesis. Lipid molecules from each of these sources can be used to generate ATP in the liver or they can be packaged along with apolipoprotein (Apo) B-100, ApoC, and ApoE into very low-density lipoproteins (VLDL) on the endoplasmic reticulum. VLDL are released into the circulation and metabolized by target tissues in a manner similar to that of chylomicrons, with LPL hydrolyzing triglycerides to fatty acids that can be imported by cells. In this case, the remaining lipoprotein particle is further metabolized to low density lipoprotein (LDL), which can be taken up by many tissues via the LDL receptor (44).

**Figure 2:**
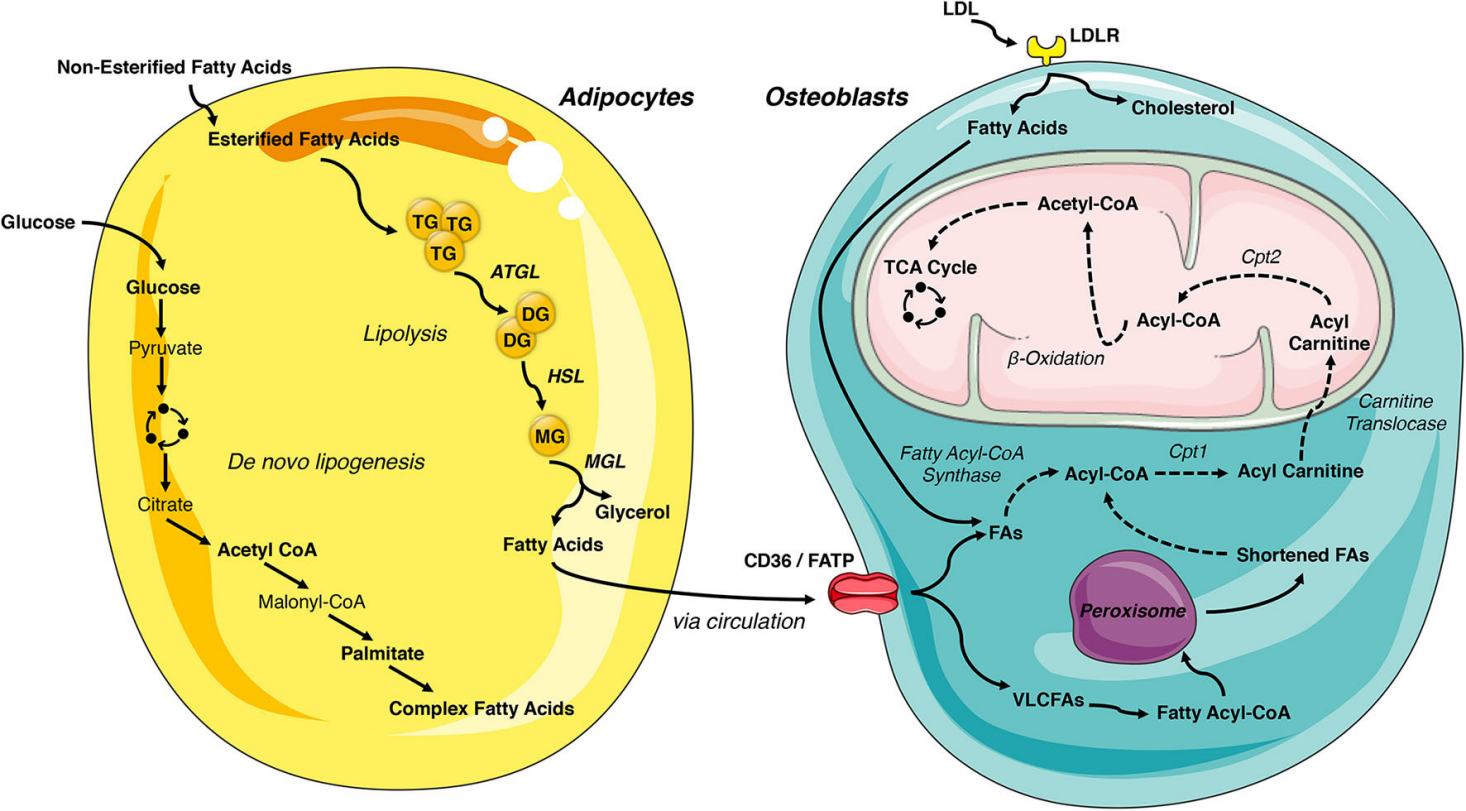
Lipid- flux between the adipocyte and osteoblast. White adipose tissue is the primary storage depot of lipids during excess consumption, which are subsequently released when energy expenditure exceeds caloric intake. Esterified fatty acids are stored in the adipocytes as triglycerides and are hydrolyzed by the rate limiting enzyme, adipose triglyceride lipase (ATGL), into diglycerides. Diglycerides are hydrolyzed into monoglycerides by hormone sensitive lipase (HSL) and further into fatty acids by monoacyglycerol lipase (MGL), which are then released into circulation. Adipocyte uptake of glucose is metabolized to acetyl-CoA and used for *de novo* fatty acid synthesis. These newly synthesized fatty acids are another lipid source for the osteoblast. LDL-derived fatty acids and uptake of circulating free fatty acids via CD36/FATPs are vital energy sources for the osteoblast. These internalized free fatty acids are converted into acyl-CoA by fatty acyl-CoA synthase. Very long chain fatty acids (VLCFAs) (more than 22 carbons) are first shortened by the peroxisome. Acyl-CoA is transported to the mitochondrial matrix by a carnitine exchange system in order to undergo β-oxidation. The product, acetyl-CoA is transferred to the TCA cycle and electron transport chain for generation of ATP. This figure was created using Servier Medical Art image templates under a Creative Commons Attribution 3.0 Unported License.

White adipose tissue is the primary storage depot for excess calories. Non-esterified fatty acids are taken up by adipocytes, esterified and stored as triglycerides, while glucose is metabolized to acetyl-CoA and then used as a substrate for *de novo* fatty acid synthesis. When energy expenditure exceeds caloric intake or in response to a number of lipolytic hormones, the stored triglycerides can be hydrolyzed to glycerol and free fatty acids that are released into the circulation to be used for β-oxidation in other organs, including the skeleton. Lipolysis is mediated by the stepwise action of three lipases (illustrated in Figure 2). The rate limiting enzyme, adipose triglyceride lipase (ATGL), catalyzes the first reaction by hydrolyzing triacylglycerols at the sn-2 position to diacylglycerol and one fatty acid. Diacylglycerides are then preferentially hydrolyzed by hormone sensitive lipase (HSL) at the sn-3 position to yield a second free fatty acid (46). Monoacyglycerol lipase (MGL) catalyzes the final reaction generating glycerol and a third fatty acid (47). Most free fatty acids released into circulation are bound by albumin (48).

The intracellular metabolism of fatty acids taken up by cells depends on chain length. Short-chain (1-6 carbons) and medium-chain (7-12 carbons) fatty acids are produced by the bacterial fermentation of dietary fiber or the ingestion of dairy products. These lipids are primarily metabolized by enterocytes or by hepatocytes and are beyond the scope of this review (47). Long-chain fatty acids (13-21 carbons) are transported into cells by specific transporters (discussed below) but have limited solubility in the cytosol. To increase solubility, trap fatty acids in the cell, and produce a high energy thioester necessary for the next steps of catabolism, long-chain fatty acyl-CoA ligases catalyze the formation of fatty acyl-CoA in a reaction that requires the hydrolysis of 1 ATP to AMP. Acyl-CoA must then be transferred to the mitochondria by a carnitine exchange system to undergo β-oxidation. Carnitine palmitoyltransferase 1 (CPT1), the first and rate-limiting step in this process, is located on the outer mitochondrial membrane and catalyzes the replacement of CoA with carnitine. Acyl-carnitines are recognized and transferred by carnitine-acylcarnitine translocase into the mitochondria matrix where carnitine palmitoyltransferase 2 (CPT2) reverses the reaction of CPT1 and regenerates Acyl-CoA. The four reaction β-oxidation process removes 2 carbons from the carboxy end of the acyl-CoA to generate acetyl-CoA, 1 NADH and 1 FADH_2_ that are transferred to the TCA cycle and electron transport chain for the generation of ATP (Figure 2). Successive rounds of β-oxidation are necessary to fully metabolize long-chain fatty acids (49).

Very long chain fatty acids (more than 22 carbons) can also be used to generate ATP but must be chain-shortened in peroxisomes before they can enter the mitochondria (50). Multi-functional peroxisomes encase more than 50 enzymes, with more than half involved in fat metabolism, in a single lipid bilayer. As in long chain fatty acid metabolism, very long chain fatty acids are first converted to acyl-CoAs in the cytosol. The fatty acyl-CoA is then transported into the peroxisome by members of the ATP binding cassette transporter D subfamily. Peroxisomal oxidation also involves four reactions but utilizes a separate set of enzymes to shorten the fatty acid chain and is not as efficient at ATP generation as there is no respiratory chain. Indeed, while the FADH_2_ produced by one round of mitochondrial β-oxidation yields 2 ATP, the electrons from FADH_2_ produced by peroxisomal oxidation are donated to oxygen to form H_2_O_2_. For this reason, chain shortened fatty acids can be shuttled to the mitochondria for further metabolism via β-oxidation.

## Mechanisms of Lipid Uptake by Osteoblasts

Although they are smaller than those evident in adipocytes, most cells contain a lipid droplet that can presumably be used to generate ATP via β-oxidation. Histological studies indicate that both mature osteoblasts and differentiating osteoblast progenitors contain stored lipid (51, 52), but these stored lipids do not appear to be a major energy source for mature osteoblast function. Kim et al. (39) ablated the expression of ATGL in cultures of calvarial osteoblasts and mature osteoblasts and osteocytes *in vivo* (Atgl^flox/flox^; Osteocalcin-Cre), which should eliminate intracellular lipolysis, but did not find a defect in either *in vitro* osteoblast performance or bone structure *in vivo*. Therefore, osteoblasts appear to require extracellular lipid sources.

A combination of *in vivo* and *in vitro* studies have examined the uptake of circulating lipoproteins and free fatty acids by the osteoblast and the skeleton. In perhaps the most comprehensive study, Neimeier and colleagues (53) modeled postprandial lipoprotein uptake by intravenously injecting fluorescent- or ^125^I-labeled chylomicron remnants into mice. Skeletal uptake was 17% that of liver but was greater than other catabolic organs including muscle and heart. Importantly, chylomicron remnant uptake by the osteoblast-/osteocyte-enriched femoral diaphysis was greater than that of bone marrow, indicating the skeletal acquisition was not simply carried out by marrow adipocytes. Osteoblasts also appear to take up of LDL and VLDL and acquisition can be enhanced by co-administration with ApoE, but these studies have primarily been performed in cultured osteoblasts (54–56). Skeletal uptake of fatty acids was assessed *in vivo* by Bartelt et al. (57) and Kim et al. (39) after delivering ^3^H-linoleic acid and ^14^C-palmitic acid or ^3^H-bromo-palmitate, respectively, via oral gavage. Similar to the uptake of chylomicron remnants, these studies revealed that skeletal acquisition of fatty acids is comparable to tissues that are more classically associated with fatty acid metabolism. Together, these studies highlight a potential role of bone in fatty acid metabolism and postprandial clearance of fat from the circulation.

The identity and requirements for specific receptors and transporters that allow osteoblasts to take up fatty acids and lipoproteins (Figure 2) need additional study, but experimental data exists for a number of possible mechanisms. Consistent with osteoblastic uptake of chylomicron remnants and lipoproteins, osteoblasts express the low-density lipoprotein receptor (LDLR) and low-density lipoprotein receptor-related protein-1 (LRP1) (58, 59). Interpretation of the skeletal phenotypes of mice engineered to be deficient for LDLR (LDLR^−/−^) requires care as studies have reported both reduced (60) and elevated bone volume (61) relative to wildtype mice. Both *in vivo* (60) and *in vitro* (62) analyses indicate that the actions of the LDLR are important for osteoblast function as its ablation results in reductions in the expression of gene markers of osteoblastic differentiation. These data accord with the ability of LDL to stimulate cell growth and sustain responsivity to anabolic stimuli in osteoblasts cultured under serum-free conditions (63). The discrepancies in bone volume observed *in vivo* are likely be related to the requirement for LDLR during osteoclast differentiation (60, 61).

LRP1 can facilitate the endocytosis of triglyceride and cholesterol containing chylomicron remnants in cultures of osteoblastic cell models (58) and polymorphisms in the gene encoding this receptor are associated with bone mineral density (64). However, analysis of an osteoblast-specific knockout mouse (Lrp1^flox/flox^; Runx2-Cre) revealed an osteopenic phenotype but there was no effect on systemic lipoprotein clearance or osteoblasts' ability to sequester fatty acids (65). While the bone phenotype has been attributed to marked increases in osteoclastogenesis (65), the sustained ability to take up lipoproteins could be due to the engagement of other LDLR family members. LRP5 and LRP6 are most typically associated with the propagation of signaling in response to Wnt ligands (66), but these receptors also have the capacity to bind and mediate the endocytosis of lipoproteins and chylomicron remnants (67, 68). Cultured osteoblasts rendered deficient for LRP5 also retained the ability to take up LDL (56), indicating that combinatorial genetic studies wherein the expression of multiple LRP receptors are simultaneously ablated may be necessary to discern receptor function in lipid particle uptake.

Osteoblasts also take up high density lipoproteins (HDL) and express Scarb1 (also referred to as SR-B1) (55), the major receptor for high-density lipoproteins (69). Some epidemiological studies suggest a positive correlation between BMD and HDL levels, but others have reported contradictory results [see (70) for a comprehensive review]. Interpretation of an association between HDL and bone mass in animal models has been equally challenging. Martineau and colleagues (71) reported that Scarb1 null mice display increases in HDL-associated cholesterol and increases in femoral bone volume and mineralization at 2 and 4 months in association with increases in osteoblast surface and bone formation rate, which suggests a detrimental effect of HDL on skeletal homeostasis. However, it remains possible that the high bone mass phenotype in these mice is due to an increase in serum adrenocorticotropin (ACTH), which has anabolic effects on osteoblasts (72, 73). Futhermore, control and Scab1 deficient osteoblasts exhibited similar levels of HDL-cholesterol uptake *in vitro* (71). A follow-up study by this same group reported that Scarb1 deletion in MSCs increased osteoblastogenesis but decreased terminal osteocyte differentiation as vertebral osteocyte density was modestly decreased in the mutant mice (74). However, a more recent study contradicted these findings and reported Scarb1 null animals to be osteopenic in the veterbrae at 16 weeks with decreases in resorption and formation markers, and diminished osteoblast differentiation markers both *in vitro* and *in vivo* (73). Here too, alterations in bone volume were attributed to dose dependent effects of ACTH on bone. Similarly, mice with impaired HDL synthesis displayed reduced bone mass and impaired differentiation (75) suggesting a necessity for HDL in osteoblast function. Further *in vivo* studies using genetic models with osteoblast specific deletions are required to further delineate Scarb1 function and a role for reverse cholesterol transport will need to be considered.

Osteoblasts also express the receptors necessary to take up and metabolize free fatty acids. CD36 is a two-transmembrane glycoprotein receptor that binds long-chain fatty acids as well as oxidized low-density lipoprotein (oxLDL) and facilitates their transport into the cell (55, 76). While direct studies of its effect on fatty acid uptake have not yet been completed, CD36 null mice exhibit a low bone mass phenotype secondary to impaired bone formation (77) that implies fatty acid uptake is essential for osteoblast function. The SLC27 family of fatty acid transport proteins (also referred to as FATP1-6), may also contribute to osteoblasts acquisition of long-chain fatty acids for oxidation (76, 78), as multiple family members are expressed by primary osteoblasts (40).

## Requirement for Fatty Acid Oxidation in Osteoblasts

The effects of specific fatty acids on the functions of the major bone cells has recently been reviewed elsewere (79). Direct examination of the requirement for fatty acid oxidation during postnatal bone acquisition and bone repair has been examined in two studies. In the first, Kim and colleagues disrupted the expression of CPT2 in mature osteoblasts and osteocytes (Cpt2^flox/flox^; Osteocalcin-Cre) (39). As noted above, CPT2 catalyzes an obligate step in fatty acid β-oxidation and was selected for ablation in this model because it is encoded by a single gene (three isoforms of *CPT1* are present in mammalian genomes). The skeletal phenotype of the mutant mice was sexually dimorphic, with male mice fed a normal chow diet exhibiting only a transient decrease in trabecular bone volume in the distal femur and L5 vertebrae at 6 weeks of age. By contrast, female mutants exhibited defects in trabecular bone volume in the distal femur and L5 vertebrae and an expansion of cortical bone tissue area at both 6 and 12 weeks. This discrepancy between sexes appears to be related to a greater ability to adjust fuel utilization in males, as male mutants exhibited an increase in femoral glucose uptake that was not evident in female mutants. The greater inhibition of osteoblast performance and inhibition of glucose uptake in CPT2 mutant osteoblasts treated with estrogen may explain the sex differences in metabolic flexibility. Interestingly, both male and female CPT2 mutants exhibited an increase in serum free fatty acid levels, which suggests that disrupting fatty acid utilization by osteoblasts and osteocytes is sufficient to alter lipid homeostasis (39).

In the second study, van Gastel et. al. (40) identified a role for fatty acid utilization during fracture healing and the specification of skeletal cell fate. During the bone healing process, endochondral ossification is initiated by periosteal progenitor cells that differentiate to chondrocytes and form an avascular, cartilaginous callus. The callus is subsequently invaded by the vasculature (80) and replaced by bone (81, 82). Blood vessels are expected to deliver the oxygen, nutrients, and growth factors necessary to drive bone formation. Through biochemical assays and the reanalysis of an existing single cell RNAseq study of skeletal progenitors (83), Van Gastel et. al. (40) reported that chondrocytes express low levels of CPT1a and high Glut1 levels as well as elevated lactate production, which suggests that glycolysis meets the chondrocyte's energy needs. On the other hand, osteoblasts expressed high levels of Glut1 and CPT1a, exhibited higher levels of oxygen consumption, and an increased ability to metabolize palmitate, indicating a reliance on fatty acid oxdiation. Importantly, knocking down the expression of CPT1a prevented the differentiation of skeletal stem cells to osteoblasts while local injection of free fatty acids during fracture repair increased the amount of bone formed in the callus and reduced the amount of cartilage. Mechanistic studies demonstrated that reduced fatty acid availability increased the activation of FOXO3, which in turn activated SOX9 and chondrogenic specification. Taken together, these studies highlight the requirement for fatty acid β-oxidation for bone-forming osteoblasts in bone repair and skeletal development.

Evidence for a role of peroxisomal lipid oxidation in bone is largely based on the phenotypes evident in patients affected by peroxisomal disorders and global knockout models. Human and mouse genetic studies have identified 14 peroxin genes (*PEX1- PEX26*) that encode proteins necessary for either the formation of peroxisomes or the transport of cargo into the organelle. Loss of function mutations in peroxin genes, which occur at a rate of ~1 in 50,000 births, result in autosomal recessive peroxisomal biogenesis disorders (PBD) that affect a number of organ systems. Individuals with more severe PBD subtypes often exhibit craniofacial anomalies, short stature, and limb length discrepancies. Less severe subtypes have been associated with reductions in bone mineral density and an increased susceptibility for non-traumatic fractures (84–86). In the mouse, hypomorphic alleles for Pex7 leads to a reduction in longitudinal growth and impaired ossification of the digits (87), while a global knockout resulted in delayed ossification at multiple skeletal sites (88). Additional mouse genetic studies will be necessary to fully delineate the role of peroxisomes in skeletal tissue maintenance and function.

## Pathways Regulating Fatty Acid Oxidation

If fatty acid metabolism is used to generate the ATP necessary for osteoblast function, then metabolic flux in this pathway should be regulated by the signals that drive bone formation. Indeed, two of the most potently anabolic pathways, Wnt signaling and parathyroid hormone signaling, appear to drive fatty acid oxidation.

### Wnt Signaling

The anabolic effects of Wnt signaling on skeletal development, repair, and homeostasis have been well-studied (89, 90), and a number of studies have now demonstrated that the pathway coordinates the intermediary metabolism of the osteoblast with the energetic demands of bone formation (38, 91–93). LRP5 and LRP6 act as co-receptors for the Frizzled receptors that propagate Wnt signals and lead to the stabilization and activation of β-catenin (66). While the osteoblast-specific ablation of either receptor (Lrp5^flox/flox^; Osteocalcin-Cre and Lrp6^flox/flox^; Osteocalcin-Cre) results in decreases in bone mineral density and vertebral trabecular bone volume (94), Frey et al. (56) found that the LRP5 mutants also exhibited increases in fat mass and serum triglycerides and free fatty acids, suggestive of a disruption in fatty acid utilization. Indeed, analysis of gene expression in cultured osteoblasts by microarray revealed that LRP5-deficient osteoblasts exhibited a downregulation of multiple genes involved in mitochondrial long-chain fatty acid β-oxidation. The effects of these changes in gene expression on β-oxidation were confirmed by examining the oxidation of oleate, which was reduced in LRP5 deficient osteoblasts when compared to control. Expression of LRP5 with a gain of function mutation (Lrp5^G171V^) in osteoblasts produced the opposite phenotype, as the transgenic mice exhibited increases in bone volume and oxidative gene expression as well as decreases in fat mass, serum triglycerides, and fatty acids.

Subsequent genetic studies revealed that Wnt-mediated regulation of fatty acid oxidation proceeds via a β-catenin-dependent mechanism. Frey et al. (95) found that only Wnt ligands that increase the abundance of β-catenin in cultured osteoblasts, increase the capacity to fully oxidize oleate to carbon dioxide. Since constitutive ablation of β-catenin in osteoblasts results in early lethality (96) *in vivo*, the generation of an inducible β-catenin knockout mouse (Ctnnb^flox/flox^; Osteocalcin-CreER^T2^) was necessary to examine the transcription factor's effects on fatty acid oxidation. In this model, the temporal ablation of β-catenin resulted in high-turnover bone loss as well as increased fat mass and the development of insulin resistance. Additionally, the expression of genes involved in long-chain fatty acid oxidation and the ability to oxidize oleate were reduced in β-catenin deficient osteoblasts *in vitro*, while serum fatty acid levels were increased in the mutants *in vivo*. These studies have expanded the role of canonical Wnt-signaling to influencing fatty acid utilization and coordinating whole-body energy homeostasis.

As indicated above, a number of contemporary studies suggest that Wnt signaling also regulates glucose and glutamine utilization by the osteoblast. Wnt signaling through LRP5 increased aerobic glycolysis in the ST2 bone marrow stromal cell line (93) and mice engineered to overexpress Wnt7b in osteoblasts exhibit dramatic increases in bone volume, but simultaneously ablating the expression of Glut1 completely inhibited the increase in bone accrual (92). Similarly, Wnt signaling induced glutamine catabolism via the TCA cycle (38) which in turn stimulated the expression of genes involved in protein sysnthesis. Interestingly, these effects were mediated by the activation of mTOR and not β-catenin (92, 93, 97). Thus, the metabolic actions of Wnt signaling appear to depend on the specific downstream pathways that are activated.

### Parathyroid Hormone Signaling

Parathyroid hormone (PTH) is a master regulator of serum calcium that signals in the bone, kidney, and intestine to increase calcium levels. Intermittent administration of human recombinant PTH (1-34) is now used to reduce the occurrence of vertebral and non-vertebral fractures and increased bone mineral density in postmenopausal osteoporotic women (98). This therapeutic effect is mediated by PTH's ability to decrease apoptosis of mature osteoblasts (99), activate preexisting bone lines cells (100, 101), and stimulate osteoprogenitor recruitment (102).

The first indication that PTH might influence fatty acid oxidation were completed by Adamek et al. (103). In this study, PTH increased palmitate oxidation in specific cell populations isolated from bone by enzymatic digestion, while 1,25-Dihydroxycholecalciferol administration produced a more dramatic effect in multiple cell fractions. A greater reliance of lipids was suggested by Catherwood et al. (63) who demonstrated that the inclusion of LDL or VLDL in a basic medium was sufficient to support the proliferative response of rat ROS17/2.8 to PTH. In a more recent work, Esen et al. (104) used Seahorse technology, radiolabeled metabolites, and MC3T3-E1 cells to examine the effect of PTH on osteoblast metabolism. These studies demonstrated that PTH stimulates glucose uptake and increases lactate production but reduces the shuttling of glucose-derived carbon to the TCA cycle. These findings suggest that the increased rate of oxygen consumption after PTH administration is due to the oxidation of another fuel source, perhaps fatty acids imported from serum. While additional studies will be necessary, this paradigm is congruent with findings from Maridas et. al (105) that tracked the transfer of fatty acids from adipocytes to bone marrow stromal cells as well as the established ability of PTH to induce lipolysis in adipocytes (106). Likewise, the reduction in marrow adipose tissue volume after intermittent PTH treatment suggests that marrow adipocytes represent an energy reserve that provides fatty acids to fuel the anabolic activity of osteoblasts (105, 107). The finding that PTH can increase bone mass even under conditions of caloric restriction suggests that the relationship between PTH activity and metabolism is more complex and worthy of further study (105).

## Skeletal Consequences of Dyslipidemia

**Figure 3:**
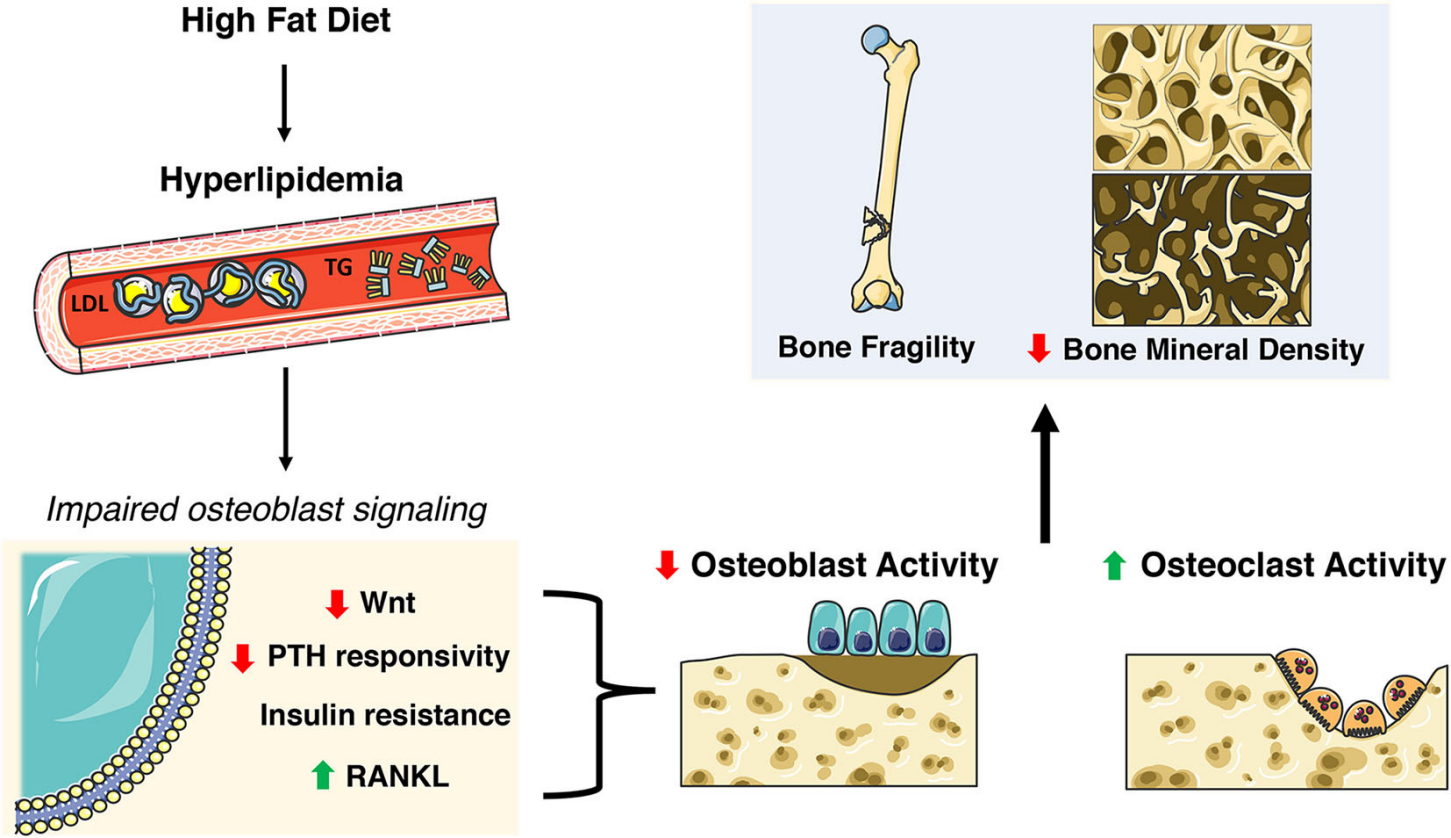
Skeletal deficits elicited by HFD-induced hyperlipidemia. A high fat diet (HFD) induces extensive systemic metabolic and skeletal changes including increases in circulating low density lipoprotein (LDL) and triglycerides (TG). This hyperlipidemic state impacts many nuances of osteoblast function and homeostasis including decreases in Wnt signaling and PTH responsiveness, insulin resistance, and increased RANKL. This results in decreased osteoblast activity and increased osteoclast activity ultimately contributing to poor skeletal health. This figure was created using Servier Medical Art image templates under a Creative Commons Attribution 3.0 Unported License.

The Centers for Disease Control (CDC) reports that 95 million adults age 20 and older have high cholesterol (>200 ng/dL) while about 25% have elevated triglyceride levels (108). The aforementioned preclinical studies suggest a requirement for fatty acid oxidation for normal skeletal development and homeostasis, but epidemiological studies suggest that dyslipidemia has detrimental effects on bone (109–114). Elevated triglycerides, hypercholesterolemia and increased LDL are associated with higher risk of osteoporosis (111, 114) while increased LDL has been associated with non-vertebral fractures (115). Likewise, the National Health and Nutrition Examination Survey (NHANES III) reports that 63% of osteoporotic patients have hyperlipidemia (116). Studies from elite endurance athletes suggest that even short term exposure to a diet rich in fat can elicit a catabolic state in bone with an increase in markers of bone resorption and decreases in bone formation markers at rest and following high-intensity exercise (117). The inverse relationship between hyperlipidemia and osteoporosis is further noted by the use of statins, a class of drugs used to lower cholesterol by blocking 3-hydroxy-3-methyl-glutaryl-CoA reductase, which was associated with an increase in BMD but no improvement in fracture risk (118–120). The sections below describe effects of dyslipidemia on osteoblast function and skeletal homeostasis in rodent models (Figure 3).

### Effect of Dyslipidemia on Bone Structure and Remodeling

Over the last decade a combination of high fat diet (HFD) feeding models and hyperlipidemic mouse models have been used to investigate the effects of dyslipidemia on skeletal homeostasis. In addition to the development of hypertriglyceridemia, these models exhibit a host of metabolic defects, including but not limited to adipose hyperplasia, hyperinsulinemia, insulin resistance, central leptin resistance, and hepatic steatosis [reviewed in (121)], that can alter the balance of bone remodeling and influence bone strength. The consensus from the majority of these studies is that HFD feeding leads to a deterioration of trabecular bone mass at multiple skeletal sites in the axial and appendicular skeleton (60, 116, 122–126). A hypercholesteremic diet produces a similar effect on trabecular bone parameters (127). Reports on the effects of HFD on cortical bone parameters are more variable. Tencerova and colleagues (125) reported that 12 weeks of HFD increased cortical porosity and decreased cortical thickness in the tibia of male C57Bl/6J mice. These phenotypes would be expected to reduce bone strength and indeed a reduction in maximum force and energy to failure were noted in the femur by Picke et al. (128) when a similar HFD feeding paradigm was employed. By contrast, Silva et al. (129) found that HFD had minimal effects on cortical bone material properties and modestly increased cortical bone area and strength in mice derived from a Large-by-Small advanced intercross, wherein inbred mouse strains with extreme body sizes were crossed. However, this study did note a discrepancy in the relationship between the expansion of femoral tissue area with increasing body mass in HFD fed mice. This finding would appear to be in agreement with the minimal effects of a HFD on cortical bone geometry in female C57Bl/6J until data were normalized to body mass (130). In all likelihood, the differences observed in the cortical bone envelope are due to the balancing of detrimental effects of metabolic dysfunction with increased mechanical loading secondary to weight gain.

Histomorphometric analyses and serum measurements of bone turnover markers consistently demonstrated that trabecular bone loss in HFD and hyperlipidemic mice is secondary to a reduction in osteoblast numbers and function as well as an increase in the abundance of osteoclasts (122, 124, 127, 131, 132). Consistent with this finding, a HFD induces a decrease in the expression of the key osteogenic transcription factors RUNX2 and OSTERIX in the bone (60, 124, 132, 133) and impairments in proliferation and colony forming capacity in bone marrow-derived mesenchymal stem cells (BM-MSCs) (125). Additionally, osteoclast precursors isolated from HFD fed mice exhibit an increased ability to form TRAP-positive osteoclasts after treatment with M-CSF and RANKL (134). The extensive effects of high fat intake were further revealed in gene expression profiling experiments performed by You et al. (135). In this study, 3 months of a high fat/high cholesterol diet led to the down-regulation of 2,200 genes and the up-regulation of 992 genes in RNA samples isolated from whole femur. Downregulated genes were implicated in a number of pathway associated with bone formation including the TGF-ß/BMP2 pathway and the Wnt pathway, while up-regulated genes were associated with the control of bone resorption. Strikingly, comparative cluster analysis of these data with changes in gene expression in ovariectomized rats, a model of osteoporosis, revealed the co-regulation of more than 1,300 genes, suggestive of a convergence of pathogenic pathways.

To dissect the effects of altered lipid metabolism from other metabolic derangements in these models, *in vitro* culture systems wherein cultures of primary osteoblasts or osteoblast-like cell lines are treated with exogenous lipids have proven helpful (124, 135–139). The common finding in these studies is that the exposure to sufficient quantities of cholesterol, palmitate or oxidized LDL [a product of LDL interaction with reactive oxygen species (140)] reduces the proliferation of osteoblastic cells, induces cell death, and impairs osteoblast differentiation. These same stimuli induce an increase in the expression of RANKL by osteoblasts and enhance osteoclastic differentiation (127). Together these studies suggest that elevated lipid levels or the presence of oxidized lipids alone are sufficient to diminish osteoblast function and in turn lead to an imbalance in anabolic and catabolic processes in the skeleton.

## Hyperlipidemia'S Impact on Anabolic Pathways of the Osteoblast

The precise mechanisms by which exogenous or oxidized lipids impair osteoblast function are not completely understood. One potential explanation is the development of an inflammatory state that is thought to contribute to metabolic dysfunction in other tissues. In support of this idea, genetic ablation of the inflammatory cytokine TNFα inhibits bone loss associated with a HFD and the detrimental effects of palmitate on osteoblast differentiation (124). Additionally, a dual impact of lipids on inflammation has been noted. While polyunsaturated omega-3 fatty acids are thought to be beneficial to bone health (79, 141–144), and have anti-inflammatory affects (145), omega-6 fatty acids have been reported to be pro-inflammatory (146), leading to pathological bone remodeling and contributing to bone fracture and osteoporosis (79). In addition to inflammatory effects, a combination of *in vitro* and *in vivo* evidence suggests that dyslipidemia desensitizes osteoblasts to anabolic stimuli, including those that regulate lipid utilization.

### Wnt Signaling

In additional to regulating the utilization of fatty acids by osteoblasts, Wnt/β-catenin signaling is vulnerable to the detrimental effects of HFD feeding. At the most proximal end of the signaling pathway, dyslipidemia appears to result in an increase in the expression of several secreted antagonists of Wnt signaling. Increases in the abundance of both Dkk1 and Sclerostin in serum have been reported in mice fed a HFD, while the latter was also found to be increased in the serum of *ob/ob* and *db/db* mice (128, 147–149). Similar increases have also been noted in obese humans and were accompanied by increases in Dkk-2 and secreted Frizzled-related proteins (150). At the distal end of the pathway, obesity and high fat diet feeding were associated with a reduction in β-catenin protein levels in the femur (151, 152). In a more extreme example, HFD feeding of the ApoE^−/−^ atherosclerosis mouse model, which induces marked decreases in osteoblast numbers and an inhibition of bone formation, resulted in widespread reductions in the expression of Wnt ligands and target genes at multiple skeletal sites (131). The mechanisms underlying these changes in transcription are not yet known.

Aside from changes in gene expression, Wang et al. (153) documented an interaction between the Wnt co-receptor LRP6 and oxidized phospholipids and oxidized LDL, produced as a result of an increase in reactive oxygen species. In this study, HFD fed mice exhibited consistent decreases in the numbers of osteoblast progenitors and the abundance of LRP6 at the cell surface in this cell population. Additional studies revealed that oxidized phospholipids and oxidized LDL induced the endocytosis of LRP6 and rendered cells resistant to the propagation of Wnt signaling. Considering the requirement for LRP6 function for the maintenance of normal bone mass (94), this mechanism may partially explain the ability of antibodies that neutralize oxidized phospholipids to attenuate bone loss due to a HFD (154).

### Parathyroid Hormone Signaling

As indicated earlier, supplementation of basal, serum-free medium with LDL is sufficient to rescue responsivity to PTH (63). However, an overabundance of serum lipid can attenuate intermittent PTH-induced bone formation as evidenced by studies in the hyperlipidemic Ldlr^−/−^ and Apoe^−/−^ mouse lines. Intermittent PTH did not increase total bone mineral density or bone mineral content in the femur of these models, and PTH-induced increases in multiple parameters of trabecular bone structure were diminished or abolished in Ldlr^−/−^ mice (155). Later studies suggested that PTH resistance is likely to be due to the accumulation of oxidized lipids as administration of the D-4F peptide, which reduced lipid oxidation products, restored the anabolic effect of PTH (156–158). Given the requirement for LRP6 for normal PTH signaling (157, 159), resistence to PTH may also be mediated by oxidized LDL-induced internalization of LRP6 (157).

### Insulin

The importance of insulin signaling in the osteoblast is revealed by the increased risk of fracture and decreased BMD in type 1 diabetes [reviewed in (160–162)], increased fracture risk despite an increase in BMD in type 2 diabetes (163, 164), and studies utilizing genetic mouse models in which insulin receptor expression is manipulated. The latter demonstrates that insulin receptor signaling is required for proliferation, survival, and osteoblast differentiation, as well as the ability of the osteoblast to contribute to the regulation of whole-body metabolism (165–167). As in skeletal muscle and adipose, dyslipidemia appears to lead to insulin resistance in the osteoblast. Wei and colleagues (168) demonstrated that mice fed a HFD exhibited reduced IRS1/2 phosphorylation in osteoblasts after insulin stimulation *in vivo* and that stearate treatment *in vitro* led to SMURF-mediated ubiquitination of the insulin receptor. HFD did not reduce trabecular bone volume in this study (perhaps due to a reduced number of osteoclasts), but multiple markers of bone formation were reduced which suggests that skeletal insulin resistance may contribute to bone loss associated with dyslipidemia.

## Peroxisome Proliferator-Activated Receptor γ (PPARγ)

A final mechanism by which hyperlipidemia could impact osteoblast performance and skeletal homeostasis is through the activation of *PPAR*γ, a transcriptional regulator of adipogenesis that can be activated by elevated lipid levels. In bone, the nuclear receptor influences bone remodeling by stimulating adipogenic differentiation of mesenchymal stem cell at the expense of osteoblastogenesis and by stimulating osteoclastogenesis (169–171). HFD-fed rodents exhibit increased PPARγ gene expression likely leading to defects in osteoblastogenesis (124, 151). Additionally, HFD caused an increase in callus adiposity attributed to increased PPARγ expression and was associated with decreased osteoblast surface during late stages of healing post-fracture (172). One potential explanation for these finds is the ability of PPARγ to interfere with anabolic Wnt/β-catenin signaling (173–175). These effects are critically important for the targeting of PPARγ function in the treatment of type 2 diabetes. Thiazolidinediones (TZDs), synthetic PPARγ ligands, are used to increase insulin senstivity (176–178) but do so at the expense of skeletal health. Long term use of these agonists increased risk of fractures in women (179, 180) and decreased bone formation makers (181) while short term use was sufficient to decrease bone formation markers, total hip bone density, and lumbar spine bone density (182).

It is important to note that genetic ablation of PPARγ has beneficial effects on bone and body composition. Akune and colleagues (169) reported that PPAR^+/−^ exhibit an increase in trabecular bone volume secondary to a doubling of the osteoblast surface. When PPARγ expression was ablated in mature osteoblasts and osteocytes (PPARg^flox/flox^ DMP1-Cre), the mutant mice exhibited increases in femoral bone mineral density and trabecular bone volume as well as reduced fat mass and increased energy expenditure (183). Crosstalk between osteoblasts and adipocytes in this model was indicated by *in vitro* studies wherein the 3T3-L1 adipocyte cell line was treated with medium conditioned by PPARγ deficient osteoblasts culture media and exhibited reduced Oil Red O staining than those exposed to medium conditioned by wildtype osteoblasts (183). Furthermore, PPARγ ablation in mature osteoblast/osteocytes protected against HFD-induced metabolic affects by improving liver steatosis, increasing lean mass, preventing fat mass increases, maintaining wild-type glycemic control, and improved biomechanical strength (183). Therefore, modulating PPARγ's function in the osteoblast could be a potential target for combating bone loss associated with hyperlipidemia.

## Concluding Remarks

In this review, we have attempted to convey the necessity of lipid utilization by the osteoblast for normal skeletal homeostasis as well as the potential for dyslipidemia to impair osteoblast function and lead to an imbalance in bone remodeling. Mitochondrial long chain fatty acid oxidation is of sufficient importance for osteoblast function that [1] genetic impairments in this metabolic pathway lead to alterations in whole body lipid homeostasis and [2] signaling pathways essential to bone mass accrual influence fatty acid metabolism. Future studies should be directed toward more fully delineating the mechanisms of fatty acquisition by osteoblasts. These studies will require the development of new genetic mouse models in which transporters are disrupted specifically in the osteoblasts as global knockout models exhibit disturbances in metabolism that may indirectly influence bone remodeling. Determining the mechanisms by which osteoblasts convey their need for sufficient fatty acid supply to other tissues is equally vital. In this regard, the emergence of bone as a hormone-producing tissue is likely to provide key insights into the responsible endocrine networks. As we noted above, the detrimental effects of dyslipidemia, particularly in response to a high fat feeding in rodent models, on bone mass and the balance of bone formation and resorption are well-known, but the underlying mechanisms are still poorly understood. The increased recognition of bone as a lipid-utilizing tissue is likely to lead to a renewed interest in this area. Together these studies will provide a deeper understanding of the intimate interaction between the skeleton and metabolism and hopefully lead to treatment strategies that simultaneously reduce the burden of obesity and metabolic disease and preserve skeletal homeostasis.

## Author Contributions

All authors contributed to the drafting of this manuscript and approved the final version.

## Conflict of Interest

The authors declare that the research was conducted in the absence of any commercial or financial relationships that could be construed as a potential conflict of interest.
